# Rhinitis

**Published:** 1890-01-18

**Authors:** 


### SURGERY.

### RHINITIS.

In the *New York Medical Record* for Dec. 3rd last Dr. Thomas R. Rumbold gives five reasons for failure in treating chronic rhinitis. The first reason is "defective instrumentation." The blades of the tongue depressors used are too wide and too long, and the patient should be allowed to hold it himself. Secondly, spray producers used are made for watery solutions, but an instrument which will throw warm vaseline is required. Thirdly, compressed air used is taken from a container of a capacity of from three to four or five gallons, which is three times greater than that which should be employed. Then the instrument, being composed of brass and decomposing leather, must harbour bacteria. The fourth reason for failure is an anatomical one. Rhinal inflammation commences on the superior and middle turbinated processes, and extends from thence. Anterior nasal treatment is not applicable. The only instruments useful are the posterior nares syringe and black rubber spray producer. Dr. Rumbold's fifth reason is the etiological one. Inflammation is due to an enlargement of the bloodvessels and increase in the number carrying red blood. Cold, tobacco, alcohol, carbolic acid, etc., act as irritants. The indications, therefore, are to remove diseased secretion which is always acrid ; to prevent new secretion becoming acrid ; to remove causes of irritation. But while condemning instruments used, and local applications used, as tending to create rather than to lessen irritation, the writer has no precise rules instead. Now, in our experience chronic rhinitis usually depends on some constitutional defect. It may be tubercular, syphilitic, gouty, or it may depend on polypi or other growths. Local treatment, when rhinitis depends on a constitutional cause, is, we consider, of secondary importance. The author, however, does not say anything on this subject.

